# The Annual Report of the National Board of Health

**Published:** 1883-01

**Authors:** 


### THE ANNUAL REPORT OF THE NATIONAL BOARD OF HEALTH.

This document, of forty-three pages, reviews the history of the Board from its inception to the present time. It details the efforts the Board has made to carry out the true spirit of the Act calling it into existence, and strenu ously denies the charges of extravagance which, it seems, have been preferred, and which, it appears, were the principal cause of the recent adverse legislation on the part of Congress in withdrawing the necessary and usual ap propriations.

It recommends that Congress should secure the collection of trustworthy vital statistics by authorizing the expenditure of twenty-five thousand dollars for this purpose, as, it is claimed, the data supplied by these returns furnishes the basis of all real sanitary improvement, and the amount asked for is considered very small in comparison with the benefits which it is believed will accrue.

The Board, in this report, deplores the forced suspension of the *Bulletin*, as it had become an acceptable and important means of distributing a kind of information not satisfactorily distributed by any other publication, and it recommends its re-establishment.

The charge that the Board was constituted with special reference to the occurrence of cholera and yellow fever, and that these reasons for its creation having ceased, it, by continuing to discharge its functions, transcends the limits of authority prescribed by law, is scarcely worthy of consideration, nevertheless it is refuted by the argument of the Board bearing upon this asseveration.

Altogether the National Board, or, as it should be called, the Board of Health of the United States of America, makes out a strong case in its favor and, indeed, nothing less could have been expected, for the strength of a sound public policy rests with the Board, and we hope soon to see it re-habililitated and again engaged in the exercise of the responsible duties which, under wisely adjusted regulations, will necessarily devolve upon it.

